# Testing the proportional hazards assumption in cox regression and dealing with possible non-proportionality in total joint arthroplasty research: methodological perspectives and review

**DOI:** 10.1186/s12891-021-04379-2

**Published:** 2021-05-28

**Authors:** Ilari Kuitunen, Ville T. Ponkilainen, Mikko M. Uimonen, Antti Eskelinen, Aleksi Reito

**Affiliations:** 1 Mikkeli Central Hospital, Porrassalmenkatu, 35-37 50100 Mikkeli, Finland; 2 University of Eastern Finland, School of Medicine, Yliopistonranta 1, 70210 Kuopio, Finland; 3 Department of Orthopedics and Traumatology, Central Finland Hospital Nova, Keskussairaalantie 19, 40620 Jyväskylä, Finland; 4 COXA Hospital for Joint Replacement and Faculty of Medicine and Health Technologies, Tampere University, Niveltie 4, 33520 Tampere, Finland; 5 Department of Orthopedics and Traumatology Tampere, Tampere University Hospital, Elämänaukio 2, 33520 Tampere, Finland

**Keywords:** Methodology, Survival analysis, Reproducibility

## Abstract

**Background:**

Survival analysis and effect of covariates on survival time is a central research interest. Cox proportional hazards regression remains as a gold standard in the survival analysis. The Cox model relies on the assumption of proportional hazards (PH) across different covariates. PH assumptions should be assessed and handled if violated. Our aim was to investigate the reporting of the Cox regression model details and testing of the PH assumption in survival analysis in total joint arthroplasty (TJA) studies.

**Methods:**

We conducted a review in the PubMed database on 28th August 2019. A total of 1154 studies were identified. The abstracts of these studies were screened for words “cox and “hazard*” and if either was found the abstract was read. The abstract had to fulfill the following criteria to be included in the full-text phase: topic was knee or hip TJA surgery; survival analysis was used, and hazard ratio reported. If all the presented criteria were met, the full-text version of the article was then read. The full-text was included if Cox method was used to analyze TJA survival. After accessing the full-texts 318 articles were included in final analysis.

**Results:**

The PH assumption was mentioned in 114 of the included studies (36%). KM analysis was used in 281 (88%) studies and the KM curves were presented graphically in 243 of these (87%). In 110 (45%) studies, the KM survival curves crossed in at least one of the presented figures. The most common way to test the PH assumption was to inspect the log-minus-log plots (*n* = 59). The time-axis division method was the most used corrected model (*n* = 30) in cox analysis. Of the 318 included studies only 63 (20%) met the following criteria: PH assumption mentioned, PH assumption tested, testing method of the PH assumption named, the result of the testing mentioned, and the Cox regression model corrected, if required.

**Conclusions:**

Reporting and testing of the PH assumption and dealing with non-proportionality in hip and knee TJA studies was limited. More awareness and education regarding the assumptions behind the used statistical models among researchers, reviewers and editors are needed to improve the quality of TJA research. This could be achieved by better collaboration with methodologists and statisticians and introducing more specific reporting guidelines for TJA studies. Neglecting obvious non-proportionality undermines the overall research efforts since causes of non-proportionality, such as possible underlying pathomechanisms, are not considered and discussed.

**Supplementary Information:**

The online version contains supplementary material available at 10.1186/s12891-021-04379-2.

## Background

Patient and implant specific survivals are the most tangible outcome measures in total joint arthroplasty (TJA) studies [1, 2]. These outcomes are often studied using survival or time-to-event analyses. In these analyses, reoperation or revision surgery due to any reason or due to some more specified reason are usually used as a failure or event variable. Although numerous survival analysis methods are available, the two most popular methods used to assess survival in TJA studies are the Kaplan-Meier (KM) method and Cox proportional hazards regression analysis [3]. The KM method is considered the gold standard for analyzing the survival of joint prostheses [4].

In addition to a time-to-failure and a crude survival rate, adjusted survival rates and the factors influencing them are topics of even greater research interest in TJA research. In time-to-event analysis, the main method used to assess effect of covariates and factors explaining variability in the hazard function is the Cox proportional hazards regression model. The model was created to estimate the relative hazard of an event in survival analysis [5]. As in statistical tests and models in general, Cox regression relies on background assumptions such as linearity and additivity of predictor variables. The fundamental assumption in the Cox model is that the hazards are proportional (PH), which means that the relative hazard remains constant over time with different predictor or covariate levels.

The PH assumption in any covariate is a strong assumption. Considering the complexity of biological and physiological responses and associations, this assumption has rarely a solid justification. Instead, hazards vary because the susceptibility of a disease varies between patients. Another source of varying and non-PH is the outcome or event definition. This scenario is especially relevant in TJA research. All-cause revision is a very common outcome or event assessed in TJA studies. As the name suggests, all different causes of revision such as infection, loosening and periprosthetic fracture are included when the all-cause revision is assessed.

The most common ways to assess the PH assumption are visual assessment of KM curves, log(−log) plots and testing of scaled Schoenfeld residuals (Supplementary 1). Violation of the PH assumption may lead to biased effect estimates in Cox regression analysis. We have provided examples of cases where the PH assumption is violated in Supplementary 1. Several methods have been introduced to deal with the non-proportionality. In stratified method the Cox model is stratified by the risk factor violating the PH assumption [6]. In time-axis division the Cox model is divided into time-intervals that fulfill the PH assumption. Another possibility is to use time-dependent coefficients [7]. Schemper’s weighted model is alternative methods to deal with PH violation [8]. Restricted mean survival time avoids the proportionality issues related to the Cox model [9–11]. However, it should be noted that in certain cases, PH violation alone does not automatically lead to biased estimates and non-proportionality is not an issue. If censoring is absent or censoring is independent of tested covariates, average hazard ratios are valid and interpretable as such.

A systematic review of oncological studies reported that the testing of the PH assumption was performed in only two of the included 28 studies [12]. Another systematic review reported that none of the 14 studies provided testing of the PH assumption of the Cox model [13]. Within TJA literature, survival analysis using Cox proportional hazards regression modeling has been probably one of the most applied statistical approaches. Inappropriate examination of the fundamental assumptions behind the method may cause considerable bias into effect estimates obtained from such models and these biased effect estimates may be utilized when making treatment guidelines.

The aim of our present study was to investigate the reporting of the Cox regression model and testing of the PH assumption in survival analysis in TJA studies. Furthermore, we evaluated how the possible non-proportionality issues have been addressed.

## Methods

The Pubmed database search was performed on August 28, 2019, and no language restrictions or time restrictions were used (Supplementary file 2). The abstracts of all the studies were first screened by the authors (IK and AR) for the words: “cox” or “hazard*”, and if either was found the abstract was read. The abstract had to fulfill the following criteria to be included in the full-text phase: topic was knee or hip replacement surgery; survival analysis was used, and hazard ratio reported. If all the presented criteria were met, the full-text version of the article was then read. The full-text was included if Cox method was used to analyze TJA survival. In case of discrepancy the authors AR and IK decided together whether the study should be included or excluded from the review and if unanimous decision was not found a second opinion was asked from author VP.

### Data collection

The following data were collected from the full-texts: year of study, author(s), journal, study design, number of patients or joints, outcome, intervention or exposure of interest, KM mentioned in the methods (yes or no), graphical interpretation of KM, crossing of survival curves in KM or in any other crude survival method used instead of KM (for example, Competing risk analysis). Outcome of the included studies was defined as death, revision surgery or any postoperative implant specific complication. All graphically presented KM curves were investigated. Crossing of KM survival curves was assessed and labeled in binary fashion.

The study methods sections were read and assessed quantitatively for mention of the PH assumption regarding the Cox model. If there was no mention about the PH assumption or proportionality of hazards in the methods section of the study, it was classified as the PH assumption unassessed. If the PH assumption was mentioned in some way, the test method was then collected. The PH assumption testing methods were classified as missing or not reported, visual inspection of log-minus-log plots, testing the correlation of scaled Schoenfeld residuals with time, test for log of time interaction, general graphical evaluation of the PH assumption based on KM curves or score-process test. Comments on either the fulfilled PH assumption or violation were searched from the whole text. Possible PH violation was also checked by the authors (IK and AR) by visually inspecting the crossing of the survival curves as mentioned above if any survival function was presented. If one or more of the presented KM curves crossed, the PH assumption was marked as suspicious. Clearly overlapping or non-diverging curves were not considered as crossing. Further, we also searched whether the violation of the PH assumption was considered and adjusted in the analysis. The correcting methods for the Cox model were classified as time-axis division, time-dependent coefficients, Schemper’s weighted regression or stratified methods.

This review has been conducted and reported according to Preferred Reporting Items for Systematic Reviews and Meta-Analyses (PRISMA) although the current review was not systematic [14]. All results were presented descriptively. The included articles are provided in the appendix along the data matrix (Supplementary file 3).

## Results

**Fig. 1:**
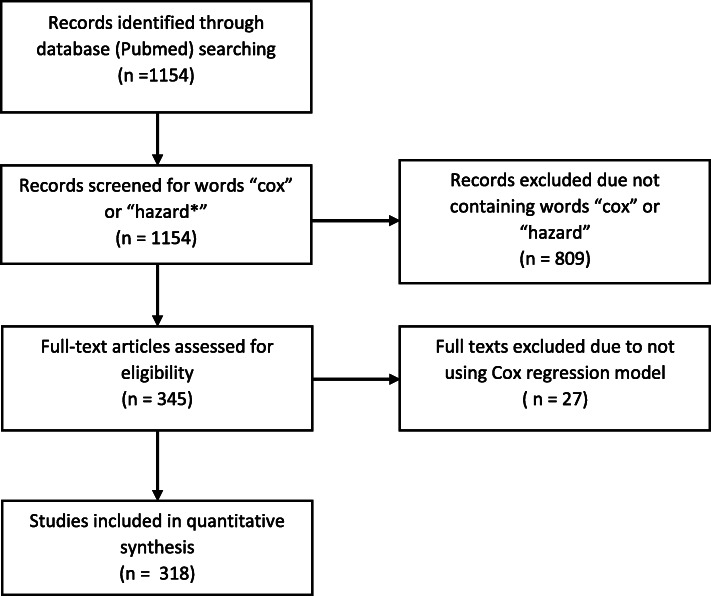
PRISMA flow diagram of the review process

A total of 1154 studies were identified by the initial search. The abstracts of these studies were screened, and 345 full-texts were read based on the abstracts. In total, 27 articles were excluded due to the use of another regression model instead of the Cox model. Finally, 318 articles were included and analyzed. (Fig. 1).

Of the included articles, 78% were register-based. The number of joints analyzed in these studies varied from 29 to 3.7 million (Table 1). To assess the crude survival, KM analysis was used in 281 (88%) studies and applied competing risk analysis as cumulative incidence function was used in the remaining 37 (12%) (Table 2). Two hundred forty-three studies reported survival probabilities graphically. Of those, 110 had crossing or non-parallel curves. In 68 studies, the KM survival curves crossed, and the PH assumption was probably violated, but the authors did not comment on the possible violation.

**Table 1:** Baseline characteristics of the included studies

	Included studies	
	*n* = 318	%
Study-design		
register-based	250	78
cohort	15	5
case series	51	16
RCT	3	1
Number of participants^a^	7170	29–3,700,000
Joint of interest		
hip	183	57
knee	114	36
both	21	7
Intervention studied		
prosthesis	170	54
operation	65	20
patient	55	17
surgeon	8	3
hospital	7	2
medication	7	2
antibiotics	6	2
Outcome measurement		
revision	300	94
death	9	3
complication	9	3

^a^median + range

**Table 2:** Method reporting in the included studies

	Included studies	
	*n* = 318	%
Assessment of proportional hazards (PH)		
Yes	127	40
No	191	60
PH testing method used		
Log(−log)	59	46
Schoenfeld residuals	42	33
Log of time	15	12
Graphical evaluation of KM curve	10	8
Score process test	1	1
Result of the PH testing state	51	40
PH violation stated	46	36
PH violation addressed in analysis	41	89
Time axis division	30	73
Time dependent coefficients	7	17
stratified analysis	4	10

Altogether 191 studies did not mention whether PH assumption was considered or tested in their analyses. When PH assumption was assessed, most common method was log-log survival plots (Table 2). However, clear result of the PH assumption testing was not always mentioned. Most common method to deal with non-proportionality was time axis division (Table 2).

Of the 318 studies included, only 63 (20%) met the following criteria: PH assumption was mentioned, PH assumption was tested, the testing method of the PH assumption was named, the result of the testing was mentioned, and the Cox regression model was corrected, if required.

## Discussion

The results of this review revealed that the fundamental assumption in Cox regression, the assessment of the PH assumption is far from optimal in TJA studies. Indeed, nearly 80% of the published TJA survival studies reported information on the proportional hazard assumption of the Cox regression inadequately. More than one fifth of the studies were found to include a probable non-proportional Cox model without the authors mentioning or adjusting it. To the best of the authors knowledge, this study is the first to evaluate the methodology and the management of common pitfalls of Cox regression model in the arthroplasty research.

Since understanding the background assumptions of the selected statistical tests is vital, it is important that these shortcomings are discussed. The problems associated with the misunderstanding and misuse of statistical methods in medical research are well known [15–17]. It seems that the use of survival analysis in TJA studies is no exception. The issue of poor reporting quality of orthopedic studies was raised in 1993 when it was noted that results were reported without a proper presentation of methods. For example, life-table analysis was not used in one third of the studies, patients at risk were not presented and survival curves were not provided [3]. Based on the results of our review, it seems that poor reporting of the methodology used still exists among the recent TJA literature.

Application of any statistical method should always include assessment of theoretical foundation from biological perspective. Assessment of proportionality should never be a simple mechanistic process. For example, if all-cause revision is the main outcome, non-proportionality is more of a rule or an expectation. In total hip arthroplasty, all-cause revision includes clinical entities such as infection and dislocation. Both of these may occur in either acute postoperative period or later period, several years after index procedure. Underlying pathomechanisms in these scenarios vary greatly. Acute infection may result from an interplay between patient comorbidities and wound related bacterial contamination whereas late infection is usually hematogenous in nature. Early dislocation is usually attributed to surgical error or failure to address the underlying tissue pathology, but late dislocation may occur due to altered pelvic posture or trauma. From this perspective it is evident that the PH assumption across different covariates such as sex, age and implant type is not justified at all.

The KM method was the most common crude survival analysis performed in the included studies and it was also presented graphically in most cases. The KM method was initially developed to analyze survival in cases where the event (for example in revision surgery) would eventually occur for every followed patient [18]. However, the KM method does not involve competing risks, and may thus overestimate the event rate and survival probability [19]. An alternative survival analysis method that is being used in arthroplasty studies is competing risk analysis, which may decrease the overestimation of the event rate in some study settings (for example when death is an important competing risk) [4, 20]. As expected, all of the studies in this review used one of the crude methods to analyze the survival time. The probable crossing or divergence of KM curves was present in many studies, but unfortunately was missed or not discussed by the majority of them.

The most common methods to examine the PH assumption used in the studies we assessed were log-minus-log plots, followed by assessment of Schoenfeld’s residuals. Nevertheless, the testing of the PH assumption was either not performed or left unreported in the majority of the studies, leading to potentially biased hazard ratio estimates, and therefore biased results. As said, neglecting obvious non-proportionality undermines the overall research efforts since possible underlying causes for non-proportionality are not considered and discussed although these causes could have considerable implications in further research. With regards to dealing with non-proportionality, time-axis division was the most used method in the analyzed studies. From these methods, time-axis division is the crudest method for dealing with non-proportionality and for presenting the results in a simple way. Interestingly, neither Schemper’s weighted model or other advanced models were used in any of the evaluated studies.

Guidelines for good reporting have been created, and it has been reported that these guidelines have improved the quality of the reporting of the methods and results in medical studies [21, 22]. On the field of TJA research, guidelines and consensus statements for the reporting of survival data in arthroplasty register studies were created and published in 2011 [4, 23]. Furthermore, the Strengthening the Reporting of Observational Studies in Epidemiology STROBE guideline has been created for observational studies and a version has also been created for cohort studies [24]. The guideline presented by the Nordic Arthroplasty Register Association is a welcome start to improving the reporting of TJA register data and could be further developed to be used as a checklist prior to submission in orthopaedic journals. After all, appropriate methods are the cornerstone of reproducible science. Inappropriate or misused methods result in irreproducible results, which may eventually have implications for patient care. In this case, biased results may lead to unsuitable implant selection and thus may deteriorate the outcome of the surgery. One possible way to improve these problems would be the routine use of statisticians as a part of the review process, which is not at present the current practice even in the highest impact medical journals [25]. It seems therefore that orthopaedic journals would benefit from the assessments provided by independent methodologists and statisticians since the statistical review process is important [26–28].

We acknowledge a few limitations in our study. First, we included solely TJA studies and the conclusions of this paper do not directly represent orthopaedic research in general. Second, we only included studies focusing on knee and hip replacements, other joint locations were excluded. This might cause slight bias as the studies focusing on other joints (elbow, shoulder, ankle) might have had even worse reporting standards for Cox regression and PH assumption, as these studies are in general more rare and have smaller sample size. Third, this study includes many studies from the same study groups, which may have caused overestimation or underestimation of the proper use of the Cox regression models. Some national and institutional registries do not provide the researchers access to the raw data and instead provide the analysis and results as requested. Therefore, the problems we have reported here, might be due the reporting quality of the register and not the authors or study group itself. However, the authors always take responsibility on the results and integrity of the data. Fourth, we only searched PubMed and no other libraries were used, which may have left some studies out from the analysis, however our study was not a systematic review and instead we focused on the estimation of statistical methods. Our results are based strictly on the reporting of the authors, and therefore it may include cases where the PH assumptions, for example, have been tested but not mentioned in the methods section. Thus, the results could be valid but not properly reported. Additionally, we estimated the proportionality of the presented methods by analyzing the crossing of the KM curves. The PH assumption may be additionally violated, although the KM curves would not cross, which may cause an underestimation of the violations of the PH assumption in the selected studies.

## Conclusions

Reporting survival analysis and testing and dealing with the assumption of proportionality of the Cox proportional hazard regression analysis in TJA studies is limited. Neglecting background assumptions of most used statistical method in TJA literature may lead to unreliable and unreplicable results. Obviously, this has implications for patient care as biased results may have altered the possible THA component selection and therefore possibly worsened the outcome of the surgery. Furthermore, improved awareness and education among scientists, reviewers and editors are needed to improve the quality of orthopaedic research. This could be achieved for example by generating guidelines for the reporting of TJA register and survival studies and these guidelines could potentially serve to improve the quality of reporting. These guidelines should focus especially on the applied biostatistical aspects such as testing and handling of relevant background assumptions for the tests used. Involving applied biostatisticians and methodologists early in the study planning and inception phase is also paramount.

## Supplementary Information

**Additional file 1: Supplementary file 1**. Testing of proportional hazards in cox regression and dealing with non-proportionality issues. **Fig. S1**.A) An example of Kaplan-Meier survival graph without an obvious proportional hazard violation. B) log(−log) plot for graph A to present the testing of the proportional hazard assumption unviolated as the curves remain parallel. **Fig. S2.** A) Survival curves start as proportional, but the hazard changes and causes the survival curves to diverge. B) The log(−log) plot shows the diverge as PH violation, since the presented graphs are unparallel. **Fig. S3.** A) Survival curves cross as the hazards is firt greated in group 2 and later in group 1. B) This leads to proportional hazard violation as the log(−log) plots cross as well. **Fig. S4**. A) The hazards and survival curves start as constant but the difference evens out later. B) The log(−log) plot reveals proportional hazard violation at the end as the curves cross.

**Additional file 2: Supplementary file 2**. Search strategy for this review.

**Additional file 3: **Data matrix of included studies for this review**.**

## Data Availability

All data generated or analyzed during this study are included in this published article and its supplementary information files.

## Abbreviations

KM: Kaplan-meier
PH: Proportional hazards
TJA: Total joint arthroplasty
