# Supplementary material for: Testing the proportional hazards assumption in cox regression and dealing with possible non-proportionality in total joint arthroplasty research: methodological perspectives and review
Source: BMC Musculoskelet Disord. 2021 May 28;22:489. doi: 10.1186/s12891-021-04379-2 (PMC8161573; doi:10.1186/s12891-021-04379-2)

Supplementary file 1:

Testing of proportional hazards in cox regression and dealing with non-proportionality issues.

Visual assessment of KM curves is a commonly used method to assess the PH assumption and assumptions of constant risk or hazard. This means plotting survival function against time. Other methods include plotting log(-log) against time or log-transformed time (1). According to the PH assumption, the survival curves should follow a similar trend without crossing regardless of method of assessment (Figure S1A). In this case the log(-log) curves follows similar trends as well and remain parallel (Figure S1B). In most cases, however, this is not the case. The hazard for different covariates can be proportional for a while and then change. Hence the survival curves will diverge (Figure S2A). The log(-log) curves in this scenario crosses and confirms the PH violation. The curves may also cross, meaning that the hazard is greater in group 2 at first, while the hazard is higher for group 1 at the end (Figure S3A), which can be seen similarly in the log(-log) plot (Figure S3B). There also might be difference between the hazards and survival curves at first, while the difference will even out later (Figure S4A). In the log(-log) plot the curves are unparallel and cross, which indicates PH violation (Figure S4B). Another visual assessment method is to check constancy of log hazard ratio against stratified follow-up times. As an alternative to visual methods, likely the most used statistical approach to test the PH assumption is to test the correlation of scaled Schoenfeld residuals with rank-ordered time. In this method, correlation of time with the residuals between the observed and expected values of covariates in each failure time-point is examined. Significant correlation of residuals with time can be interpreted as a violation of the PH assumption. The formed scatterplot can be visually inspected for the fit of the PH assumption and to detect potential outliers (2). Another statistical method to assess proportionality is to test log(time) and covariate interaction for statistical significance.

Violation of the PH assumption may lead to biased and erroneous effect estimates in Cox regression analysis if not appropriately accounted. Thus, if the PH assumption does not hold, adjustment of the model to meet the PH assumption is vital. Several methods have been introduced to deal with the non-proportionality of the Cox model. The Cox model can be stratified by the risk factor that violates the PH assumption (3). The downside of this stratification is that the effect of the selected stratification factor cannot be assessed. Another method is time-axis division where the Cox model that violates the PH assumption is divided into time-intervals that fulfill the PH assumption. One common way to correct violation of the PH assumption is to use time-dependent coefficients (4). In this method, the risk factors violating the PH assumptions are divided into time-intervals similar to time-axis division, yet the interaction between these coefficients and time is included in the same model (1, 3). Schemper´s weighted model is also one possible way to deal with PH violation (5). In Schemper’s weighted model, the events and observations are analyzed based on the follow-up instead of assuming the hazard constant over time. Since the model weights the results by time, it can be used to calculate the HRs for the whole study-period (1, 5). Another method, although methodologically completely different, that avoids the proportionality issues related to the Cox model is the restricted mean survival time method, which is based on the testing of the restricted means in survival and the reporting of the differences at preselected fixed timepoints (6-8). Restricted mean survival time method has gained increasing interest as these problems related cox method have been notified more (7,9).

It should be noted that in certain cases, PH violation alone does not automatically lead to biased regression estimates and non-proportionality is not an issue. If censoring is absent or censoring is independent of tested covariates, average hazard ratios produced by Cox regression are valid and interpretable as such. Censoring distribution, however, is almost never assessed in TJA literature. In case of non-proportionality, regression estimates may be interpreted as overall average effect during the study period as suggested recently by Stensrud and Hernan (7). This approach is suitable for RCTs where study period is usually fixed and specified and patients followed for certain time. TJA research is usually observational in nature and specific study periods such as in RCTs is not used but patients are followed as long as possible with non-random censoring distribution. Hence, the PH assumption is crucial and should not be overlooked in TJA research. As said, there is a clear biological rationale why PH is likely to often fail. Not dealing the PH assumption appropriately undermines the study purpose in TJA research. Thus, if violation of the PH assumption is detected, it should practically always be handled using one of the methods above or other applicable methods.

References

1. Ranstam J, Kärrholm J, Pulkkinen P, Mäkelä K, Espehaug B, Pedersen AB, et al. Statistical analysis of arthroplasty data. Acta Orthop. 2011 -6;82(3):253-7.

2. Schoenfeld D. Partial residuals for the proportional hazards regression model. Biometrika. 1982 /04/01;69(1):239-41.

3. Kleinbaum D, Klein M. Survival Analysis: A Self-Learning Text. 1st ed. Springer; 1996.

4. Zhang Z, Reinikainen J, Adeleke KA, Pieterse ME, Groothuis-Oudshoorn CGM. Time-varying covariates and coefficients in Cox regression models. Ann Transl Med. 2018 Apr;6(7):121.

5. Schemper M, Wakounig S, Heinze G. The estimation of average hazard ratios by weighted Cox regression. Stat Med. 2009 Aug 30,;28(19):2473-89.

6. Royston P, Parmar MKB. Restricted mean survival time: an alternative to the hazard ratio for the design and analysis of randomized trials with a time-to-event outcome. BMC Med Res Methodol. 2013 Dec 07,;13:152.

7. Stensrud MJ, Hernán MA. Why Test for Proportional Hazards? JAMA. 2020 /04/14;323(14):1401-2.

8. Royston P, Parmar MKB. The use of restricted mean survival time to estimate the treatment effect in randomized clinical trials when the proportional hazards assumption is in doubt. Stat Med. 2011 Aug 30,;30(19):2409-21.

9. Li L, Yang Z, Hou Y, Chen Z. Moving beyond the Cox proportional hazards model in survival data analysis: a cervical cancer study. BMJ Open. 2020;10(7):e033965.

Figure S1 A) An example of Kaplan-Meier survival graph without an obvious proportional hazard violation. B) log(-log) plot for graph A to present the testing of the proportional hazard assumption unviolated as the curves remain parallel.


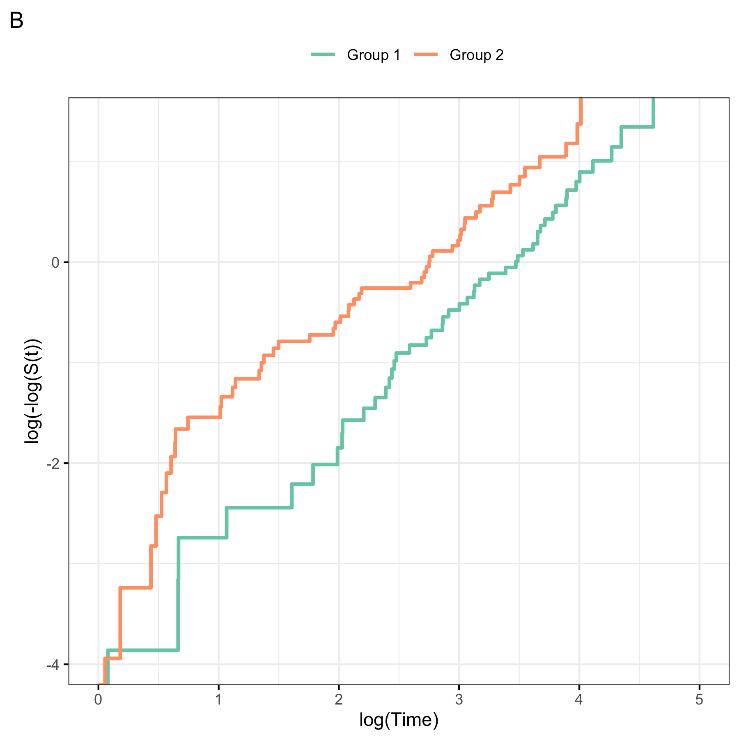

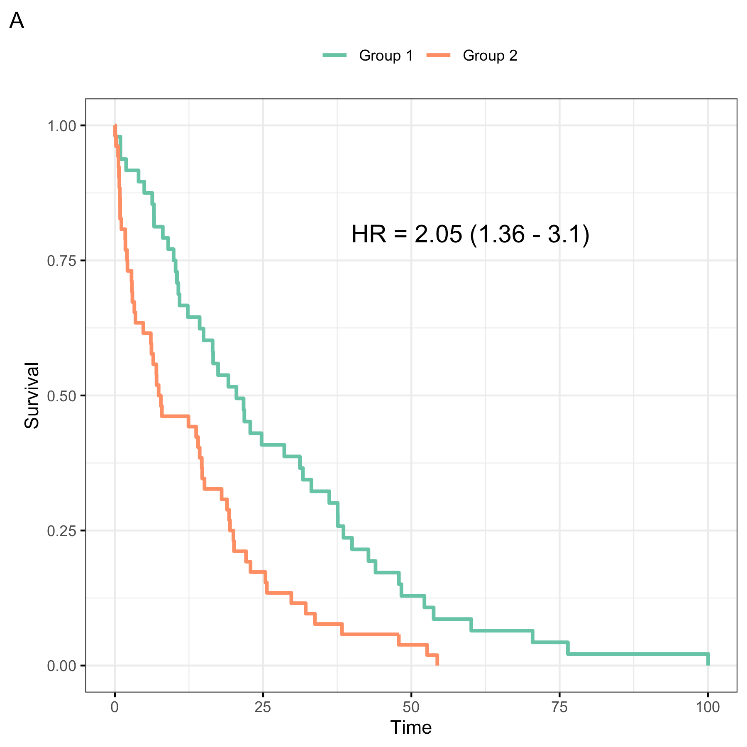


Figure S2 A) Survival curves start as proportional, but the hazard changes and causes the survival curves to diverge. B) The log(-log) plot shows the diverge as PH violation, since the presented graphs are unparallel.


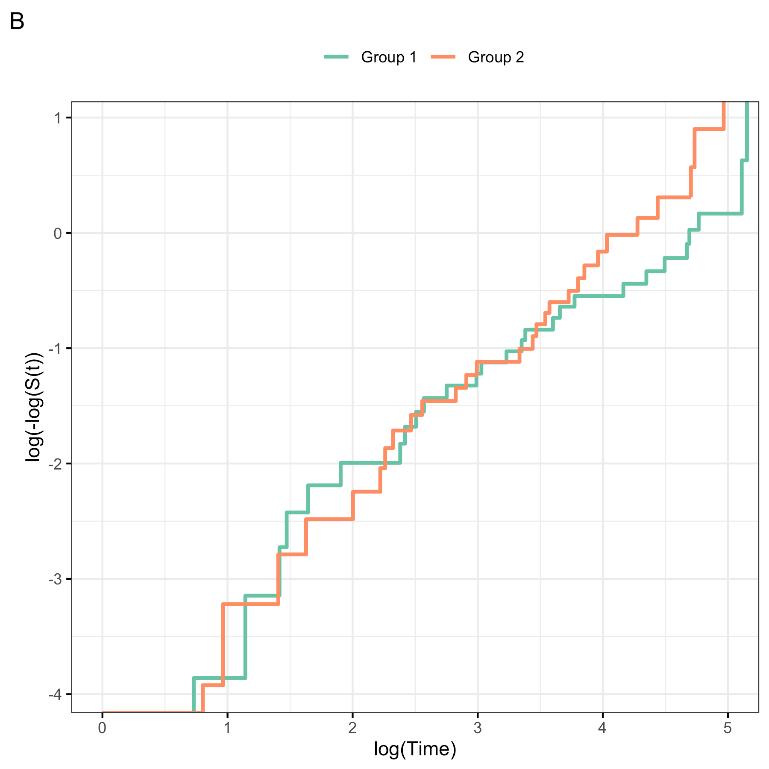

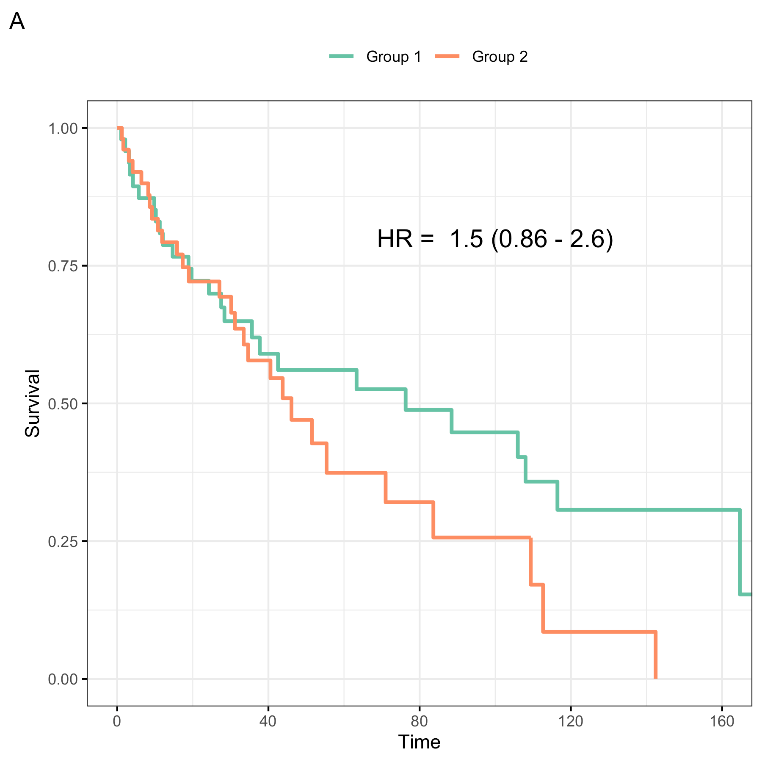


Figure S3 A) Survival curves cross as the hazards is firt greated in group 2 and later in group 1. B) This leads to proportional hazard violation as the log(-log) plots cross as well.


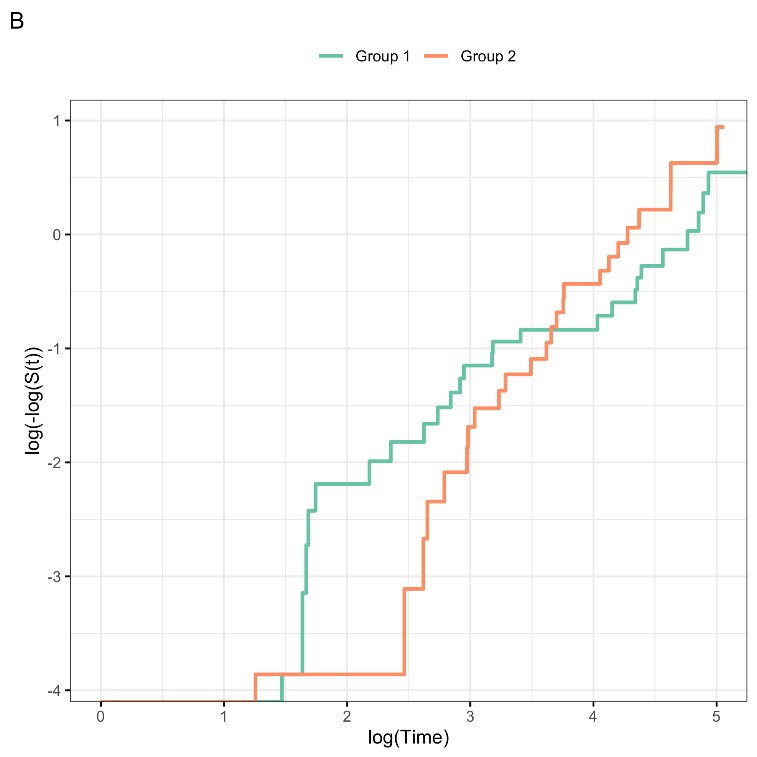

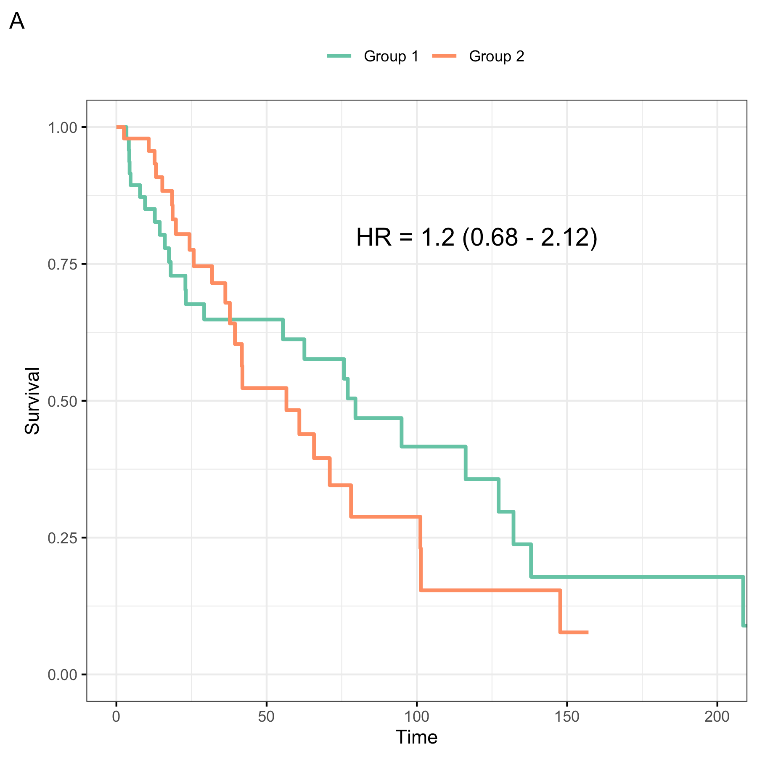


Figure S4 A) The hazards and survival curves start as constant but the difference evens out later. B) The log(-log) plot reveals proportional hazard violation at the end as the curves cross.


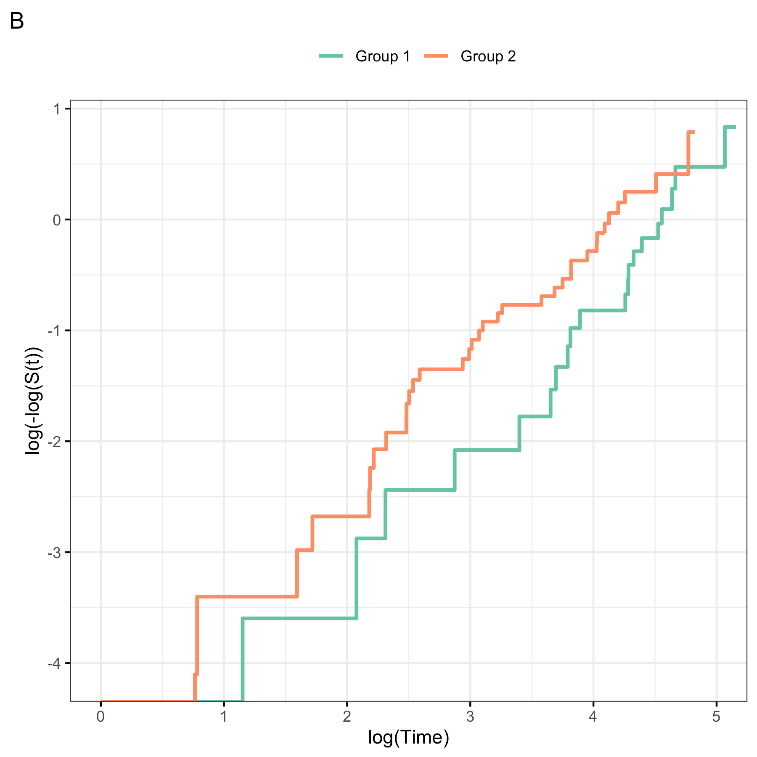

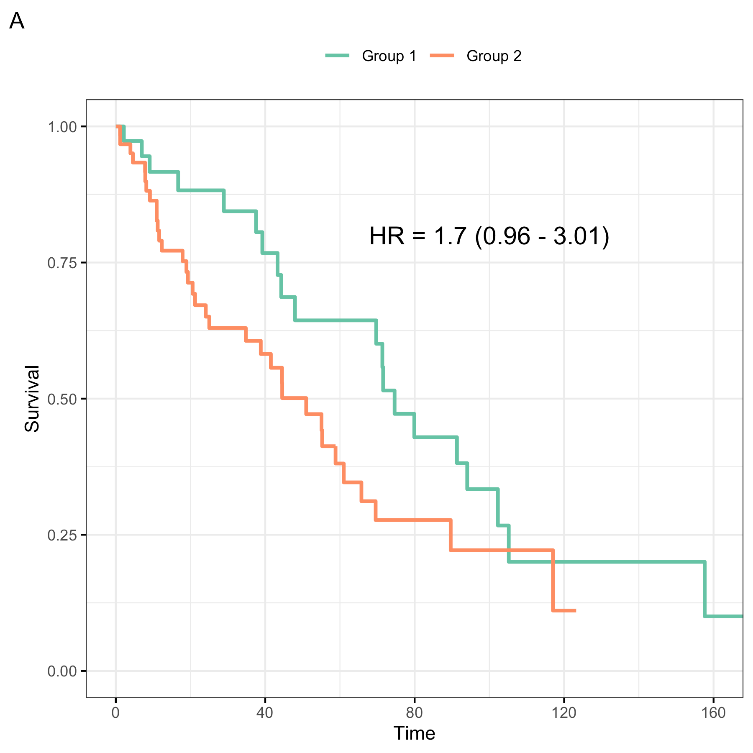

Supplement: Supplementary file 1 — Additional file 1: Supplementary file 1. Testing of proportional hazards in cox regression and dealing with non-proportionality issues. Fig. S1.A) An example of Kaplan-Meier survival graph without an obvious proportional hazard violation. B) log(−log) plot for graph A to present the testing of the proportional hazard assumption unviolated as the curves remain parallel. Fig. S2. A) Survival curves start as proportional, but the hazard changes and causes the survival curves to diverge. B) The log(−log) plot shows the diverge as PH violation, since the presented graphs are unparallel. Fig. S3. A) Survival curves cross as the hazards is firt greated in group 2 and later in group 1. B) This leads to proportional hazard violation as the log(−log) plots cross as well. Fig. S4. A) The hazards and survival curves start as constant but the difference evens out later. B) The log(−log) plot reveals proportional hazard violation at the end as the curves cross. [file 12891_2021_4379_MOESM1_ESM.docx]
