# Supplementary material for: Testing the proportional hazards assumption in cox regression and dealing with possible non-proportionality in total joint arthroplasty research: methodological perspectives and review
Source: BMC Musculoskelet Disord. 2021 May 28;22:489. doi: 10.1186/s12891-021-04379-2 (PMC8161573; doi:10.1186/s12891-021-04379-2)
Supplement: Supplementary file 2 — Additional file 2: Supplementary file 2. Search strategy for this review. [file 12891_2021_4379_MOESM2_ESM.docx]

Supplementary file 2, Search strategy for this review.

We conducted a search in the PubMed database and following search terms were used: ("arthroplasty" OR "arthroplasties" OR "replacement" OR "replacements") AND ("Knee" OR "Hip") AND ("Survival" OR "survivorship" OR "revision rate") AND ("cox" OR "risk factor" OR "risk factors" OR "hazard ratio" OR "hazard ratios").
